# Methyl 2-bromo-3-(4-chloro­benzene­sulfonamido)­benzoate

**DOI:** 10.1107/S1600536812048581

**Published:** 2013-01-31

**Authors:** Ahmad Z. Ghafoor, Brian Chang, Christopher L. King, Ray J. Butcher, Amol A. Kulkarni

**Affiliations:** a College of Pharmacy, Howard University, 2300 4th Street, NW, Washington, DC 20059, USA; b Department of Chemistry, Howard University, 525 College Street, NW, Washington, DC 20059, USA

## Abstract

In the crystal structure of the title compound, C_14_H_11_BrClNO_4_S, the mol­ecules form inversion dimers with *R*_2_^2^(8) motifs through pairs of N—H⋯O hydrogen bonds. The benzene rings are not coplanar and subtend a dihedral angle of 66.27 (8)°. The carbomethoxy group makes a dihedral angle of 75.1 (1)° with the ring to which it is attached.

## Related literature

Depending on their substitution patterns, sulfonamides display a wide array of biological activity. For their use as anti­mitotic, anti­bacterial and anti-obesity agents, see: Hu *et al.* (2008 ▶); Wydysh *et al.* (2009 ▶). For structures related to the development of novel anti­microbial agents, see: Kulkarni *et al.* (2012*a* ▶,*b* ▶).
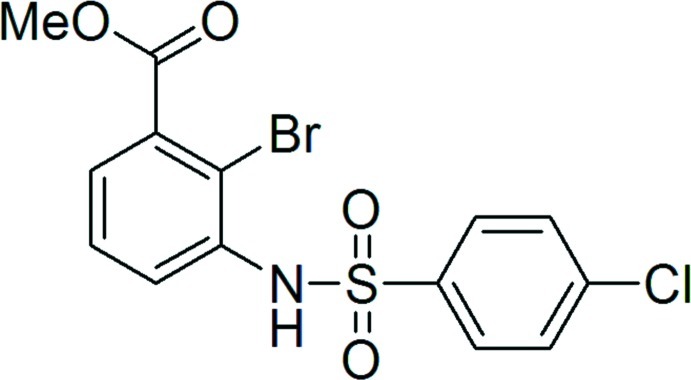


## Experimental

### 

#### Crystal data

C_14_H_11_BrClNO_4_S
*M* *_r_* = 404.66
Monoclinic, 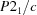

*a* = 7.9206 (2) Å
*b* = 9.4600 (3) Å
*c* = 20.0915 (6) Å
β = 94.505 (3)°
*V* = 1500.79 (8) Å^3^
*Z* = 4
Cu *K*α radiation
μ = 6.84 mm^−1^
*T* = 123 K
1.06 × 0.88 × 0.52 mm

#### Data collection

Agilent Xcalibur (Ruby, Gemini) diffractometer
Absorption correction: analytical [*CrysAlis PRO* (Agilent, 2010 ▶), based on expressions derived by Clark & Reid (1995 ▶)] *T*_min_ = 0.049, *T*_max_ = 0.198
5248 measured reflections
3012 independent reflections
2873 reflections with *I* > 2σ(*I*)
*R*_int_ = 0.033

#### Refinement

*R*[*F* ^2^ > 2σ(*F* ^2^)] = 0.034
*wR*(*F* ^2^) = 0.093
*S* = 1.08
3012 reflections
205 parameters
H atoms treated by a mixture of independent and constrained refinement
Δρ_max_ = 0.48 e Å^−3^
Δρ_min_ = −0.65 e Å^−3^

### 

Data collection: *CrysAlis PRO* (Agilent, 2010 ▶); cell refinement: *CrysAlis PRO*; data reduction: *CrysAlis PRO*; program(s) used to solve structure: *SHELXS97* (Sheldrick, 2008 ▶); program(s) used to refine structure: *SHELXL97* (Sheldrick, 2008 ▶); molecular graphics: *SHELXTL* (Sheldrick, 2008 ▶); software used to prepare material for publication: *SHELXTL*.

## Supplementary Material

Crystal structure: contains datablock(s) I, global. DOI: 10.1107/S1600536812048581/ds2221sup1.cif

Structure factors: contains datablock(s) I. DOI: 10.1107/S1600536812048581/ds2221Isup2.hkl

Supplementary material file. DOI: 10.1107/S1600536812048581/ds2221Isup3.cml

Additional supplementary materials:  crystallographic information; 3D view; checkCIF report

## Figures and Tables

**Table 1:** Hydrogen-bond geometry (Å, °)

*D*—H⋯*A*	*D*—H	H⋯*A*	*D*⋯*A*	*D*—H⋯*A*
N1—H1*A*⋯O1^i^	0.80 (4)	2.22 (4)	2.978 (3)	158 (3)

Symmetry code: (i) 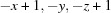.

**Fig. 1.**
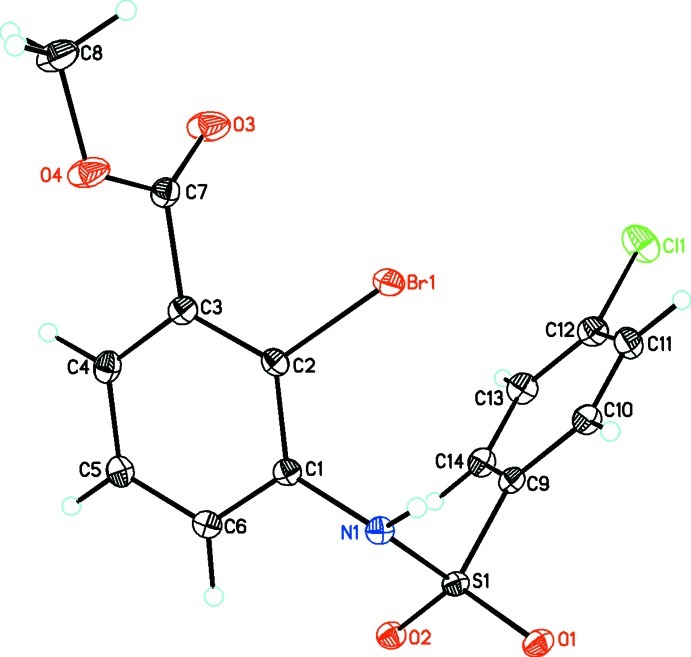
View of the molecular structure of the title compound with the atom-numbering scheme. Displacement ellipsoids are drawn at the 50% probability level for non-hydrogen atoms.

**Fig. 2.**
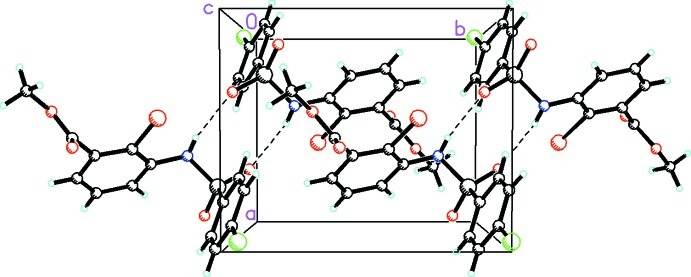
The packing view showing the hydrogen bonds network. Dashed lines indicate intermolecular N—H···O hydrogen bonds (see Table 1 for details).

## supplementary crystallographic information

### Comment

Depending on their substitution patterns, sulfonamides display a wide array of biological activity. These compounds have been used as antimitotic, antibacterial, anti-obesity agents. See: Hu *et al.* (2008); Wydysh *et al.* (2009). The crystal structure of the title compound has not previously been reported. The title compound was synthesized as an intermediate during our synthetic studies directed towards the development of novel antimicrobial agents (Kulkarni *et al.*, 2012*a*, 2012*b*).

In the title compound (C_14_H_11_Br_1_Cl_1_N_1_O_4_S_1_), the molecules form dimers through N—H···O hydrogen bonding to form *R*^2^_2_(8) motifs. The two phenyl rings are not coplanar and have a dihedral angle of 66.27 (8)°. The carbomethoxy group makes a dihedral angle of 75.1 (1)° with the ring to which it is attached.

### Experimental

A 25 ml one-neck flask equipped with a magnetic stir bar was charged with a solution of the methyl ester (230 mg, 1.0 mmole) in CH_2_Cl_2_ (4.0 ml). Pyridine (604 ml, 7.5 mmole) was added. The reaction mixture was cooled at 0 °C using ice-bath for 15 minutes. 4-chlorophenylsulfonyl chloride (253 mg, 1.2 mmole) was added to the reaction mixture. The reaction mixture was slowly warmed to RT and was stirred at RT for 4 h. The crude reaction mixture was poured into a separatory funnel containing 1 N HCl (20 ml) and CH_2_Cl_2_ (20 ml). The layers were separated, the organic layer was washed with water (2X 20 ml) and brine (1X 20 ml). It was dried over anhydr. Na_2_SO_4_. The solvent was evaporated *in vacuo*. The orange/brown oil thus obtained was purified using silica gel flash column chromatography. Elution with 40% EA in hexanes afforded the desired sulfonamide product as pale yellow crystals. mp 121–124 °C; ^1^H-NMR (CDCl_3_) d 7.80 (dd, *J* = 8.0, 2.0 Hz, 1H), 7. 68 (dt, *J* = 8.0, 2.0 Hz, 1H), 7.51 (dd, *J* = 8.0, 2.0 Hz, 1H), 7.41–7.31 (m, 4H), 3.86 (s, 3H). ^13^C-NMR (CDCl_3_) d 165.9, 139.9, 137.1, 135.5, 133.4, 129.4, 128.6, 128.0, 127.7, 125.4, 115.1, 52.6.

### Refinement

The amine H atom was seen in a difference Fourier map and refined isotropically with *U*_iso_(H) = 1.2*U*_eq_(N). The C-bound H-atoms were positioned geometrically with C—H = 0.95 and 0.98 Å, for aromatic and CH_3_ H-atoms, respectively, and constrained to ride on their parent atoms, with *U*_iso_(H) = 1.2*U*_eq_(C) [*U*_iso_(H) = 1.5*U*_eq_(C) for CH_3_].

### Crystal data

**Table:**

C_14_H_11_BrClNO_4_S	*F*(000) = 808
*M**_r_* = 404.66	*D*_x_ = 1.791 Mg m^−^^3^
Monoclinic, *P*2_1_/*c*	Cu *K*α radiation, λ = 1.54184 Å
Hall symbol: -P 2ybc	Cell parameters from 3046 reflections
*a* = 7.9206 (2) Å	θ = 4.4–75.7°
*b* = 9.4600 (3) Å	µ = 6.84 mm^−^^1^
*c* = 20.0915 (6) Å	*T* = 123 K
β = 94.505 (3)°	Chunk, colorless
*V* = 1500.79 (8) Å^3^	1.06 × 0.88 × 0.52 mm
*Z* = 4	

### Data collection

**Table:**

Agilent Xcalibur (Ruby, Gemini) diffractometer	3012 independent reflections
Radiation source: Enhance (Cu) X-ray Source	2873 reflections with *I* > 2σ(*I*)
Graphite monochromator	*R*_int_ = 0.033
Detector resolution: 10.5081 pixels mm^-1^	θ_max_ = 75.9°, θ_min_ = 4.4°
ω scans	*h* = −9→9
Absorption correction: analytical [*CrysAlis PRO* (Agilent, 2010), based on expressions derived by Clark & Reid (1995)]	*k* = −11→7
*T*_min_ = 0.049, *T*_max_ = 0.198	*l* = −24→25
5248 measured reflections	

### Refinement

**Table:**

Refinement on *F*^2^	Secondary atom site location: difference Fourier map
Least-squares matrix: full	Hydrogen site location: inferred from neighbouring sites
*R*[*F*^2^ > 2σ(*F*^2^)] = 0.034	H atoms treated by a mixture of independent and constrained refinement
*wR*(*F*^2^) = 0.093	*w* = 1/[σ^2^(*F*_o_^2^) + (0.0601*P*)^2^ + 0.5274*P*] where *P* = (*F*_o_^2^ + 2*F*_c_^2^)/3
*S* = 1.08	(Δ/σ)_max_ = 0.001
3012 reflections	Δρ_max_ = 0.48 e Å^−^^3^
205 parameters	Δρ_min_ = −0.65 e Å^−^^3^
0 restraints	Extinction correction: *SHELXL97* (Sheldrick, 2008), Fc^*^=kFc[1+0.001xFc^2^λ^3^/sin(2θ)]^-1/4^
Primary atom site location: structure-invariant direct methods	Extinction coefficient: 0.0021 (3)

### Special details

**Table:**

Experimental. CrysAlisPro (Agilent, 2010) Analytical numeric absorption correction using a multifaceted crystal model based on expressions derived by R.C. Clark & J.S. Reid. (Clark, R. C. & Reid, J. S. (1995). Acta Cryst. A51, 887-897)
Geometry. All e.s.d.'s (except the e.s.d. in the dihedral angle between two l.s. planes) are estimated using the full covariance matrix. The cell e.s.d.'s are taken into account individually in the estimation of e.s.d.'s in distances, angles and torsion angles; correlations between e.s.d.'s in cell parameters are only used when they are defined by crystal symmetry. An approximate (isotropic) treatment of cell e.s.d.'s is used for estimating e.s.d.'s involving l.s. planes.
Refinement. Refinement of *F*^2^ against ALL reflections. The weighted *R*-factor *wR* and goodness of fit *S* are based on *F*^2^, conventional *R*-factors *R* are based on *F*, with *F* set to zero for negative *F*^2^. The threshold expression of *F*^2^ > σ(*F*^2^) is used only for calculating *R*-factors(gt) *etc*. and is not relevant to the choice of reflections for refinement. *R*-factors based on *F*^2^ are statistically about twice as large as those based on *F*, and *R*- factors based on ALL data will be even larger.

### Fractional atomic coordinates and isotropic or equivalent isotropic displacement parameters (Å^2^)

**Table:**

	*x*	*y*	*z*	*U*_iso_*/*U*_eq_	
Br1	0.55830 (3)	0.29987 (2)	0.362077 (11)	0.02554 (13)	
Cl1	0.01337 (10)	−0.03081 (9)	0.18862 (3)	0.0433 (2)	
S1	0.23124 (6)	0.09272 (5)	0.48834 (2)	0.01812 (15)	
O1	0.3147 (2)	−0.03081 (17)	0.51690 (8)	0.0235 (3)	
O2	0.0901 (2)	0.15202 (17)	0.51868 (8)	0.0230 (3)	
O3	0.4253 (3)	0.6005 (2)	0.27820 (9)	0.0397 (5)	
O4	0.5888 (3)	0.7061 (2)	0.35913 (9)	0.0352 (5)	
N1	0.3799 (3)	0.2130 (2)	0.48786 (10)	0.0193 (4)	
H1A	0.466 (5)	0.177 (3)	0.4775 (17)	0.027 (8)*	
C1	0.3423 (3)	0.3532 (2)	0.46643 (10)	0.0185 (4)	
C2	0.4146 (3)	0.4121 (2)	0.41128 (10)	0.0186 (4)	
C3	0.3853 (3)	0.5527 (2)	0.39355 (11)	0.0206 (4)	
C4	0.2772 (3)	0.6340 (2)	0.42946 (12)	0.0240 (4)	
H4A	0.2549	0.7294	0.4170	0.029*	
C5	0.2022 (3)	0.5763 (2)	0.48325 (12)	0.0229 (4)	
H5A	0.1269	0.6315	0.5070	0.027*	
C6	0.2372 (3)	0.4374 (3)	0.50239 (11)	0.0208 (4)	
H6A	0.1890	0.3995	0.5404	0.025*	
C7	0.4666 (3)	0.6198 (2)	0.33630 (11)	0.0219 (4)	
C8	0.6814 (4)	0.7813 (3)	0.31100 (14)	0.0354 (6)	
H8A	0.7891	0.8150	0.3326	0.053*	
H8B	0.6143	0.8621	0.2936	0.053*	
H8C	0.7035	0.7177	0.2742	0.053*	
C9	0.1676 (3)	0.0580 (2)	0.40362 (11)	0.0203 (4)	
C10	0.2808 (3)	−0.0105 (2)	0.36475 (12)	0.0233 (4)	
H10A	0.3899	−0.0373	0.3835	0.028*	
C11	0.2321 (3)	−0.0390 (3)	0.29849 (12)	0.0277 (5)	
H11A	0.3065	−0.0871	0.2714	0.033*	
C12	0.0727 (3)	0.0038 (3)	0.27216 (12)	0.0278 (5)	
C13	−0.0388 (3)	0.0736 (3)	0.31041 (13)	0.0279 (5)	
H13A	−0.1467	0.1028	0.2913	0.034*	
C14	0.0093 (3)	0.1003 (2)	0.37711 (12)	0.0243 (5)	
H14A	−0.0659	0.1473	0.4043	0.029*	

### Atomic displacement parameters (Å^2^)

**Table:**

	*U*^11^	*U*^22^	*U*^33^	*U*^12^	*U*^13^	*U*^23^
Br1	0.03114 (18)	0.02542 (18)	0.02113 (17)	0.00412 (8)	0.00890 (10)	0.00167 (8)
Cl1	0.0431 (4)	0.0583 (4)	0.0263 (3)	0.0072 (3)	−0.0109 (3)	−0.0104 (3)
S1	0.0171 (3)	0.0196 (3)	0.0177 (3)	0.00109 (18)	0.00194 (18)	0.00302 (17)
O1	0.0231 (8)	0.0229 (8)	0.0248 (8)	0.0032 (6)	0.0033 (6)	0.0067 (6)
O2	0.0198 (7)	0.0256 (8)	0.0242 (8)	0.0008 (6)	0.0048 (6)	0.0020 (6)
O3	0.0456 (11)	0.0525 (12)	0.0200 (8)	−0.0195 (10)	−0.0034 (8)	0.0039 (8)
O4	0.0380 (10)	0.0442 (12)	0.0223 (9)	−0.0218 (9)	−0.0036 (7)	0.0062 (7)
N1	0.0168 (9)	0.0212 (9)	0.0197 (9)	0.0014 (7)	0.0005 (7)	0.0013 (7)
C1	0.0165 (9)	0.0217 (10)	0.0165 (9)	−0.0002 (8)	−0.0034 (7)	0.0007 (8)
C2	0.0168 (9)	0.0219 (10)	0.0167 (9)	0.0008 (8)	−0.0012 (7)	−0.0017 (8)
C3	0.0189 (10)	0.0230 (10)	0.0191 (10)	−0.0012 (8)	−0.0034 (8)	0.0013 (8)
C4	0.0238 (11)	0.0201 (10)	0.0271 (11)	0.0016 (8)	−0.0042 (9)	0.0010 (8)
C5	0.0192 (10)	0.0237 (11)	0.0257 (11)	0.0024 (8)	0.0008 (8)	−0.0030 (8)
C6	0.0175 (10)	0.0248 (10)	0.0199 (10)	0.0000 (8)	0.0003 (8)	0.0000 (8)
C7	0.0226 (10)	0.0212 (10)	0.0215 (10)	0.0010 (8)	−0.0015 (8)	0.0020 (8)
C8	0.0363 (14)	0.0401 (14)	0.0296 (13)	−0.0140 (11)	0.0019 (11)	0.0088 (11)
C9	0.0208 (10)	0.0186 (10)	0.0215 (10)	−0.0017 (8)	0.0005 (8)	0.0024 (8)
C10	0.0207 (10)	0.0244 (10)	0.0243 (11)	0.0018 (8)	−0.0008 (8)	0.0013 (8)
C11	0.0286 (12)	0.0285 (11)	0.0258 (12)	0.0039 (10)	0.0019 (9)	−0.0033 (9)
C12	0.0281 (12)	0.0307 (12)	0.0238 (11)	−0.0029 (9)	−0.0042 (9)	−0.0002 (9)
C13	0.0214 (11)	0.0315 (12)	0.0297 (12)	0.0002 (9)	−0.0054 (9)	0.0010 (9)
C14	0.0206 (10)	0.0244 (11)	0.0276 (11)	0.0017 (8)	−0.0001 (9)	0.0014 (9)

### Geometric parameters (Å, º)

**Table:**

Br1—C2	1.892 (2)	C4—H4A	0.9500
Cl1—C12	1.738 (3)	C5—C6	1.391 (3)
S1—O2	1.4294 (16)	C5—H5A	0.9500
S1—O1	1.4397 (16)	C6—H6A	0.9500
S1—N1	1.638 (2)	C8—H8A	0.9800
S1—C9	1.767 (2)	C8—H8B	0.9800
O3—C7	1.201 (3)	C8—H8C	0.9800
O3—Br1^i^	3.4022 (19)	C9—C14	1.383 (3)
O4—C7	1.320 (3)	C9—C10	1.394 (3)
O4—C8	1.446 (3)	C10—C11	1.383 (3)
N1—C1	1.418 (3)	C10—H10A	0.9500
N1—H1A	0.80 (4)	C11—C12	1.390 (4)
C1—C6	1.394 (3)	C11—H11A	0.9500
C1—C2	1.402 (3)	C12—C13	1.383 (4)
C2—C3	1.392 (3)	C13—C14	1.387 (4)
C3—C4	1.393 (3)	C13—H13A	0.9500
C3—C7	1.502 (3)	C14—H14A	0.9500
C4—C5	1.386 (3)		
			
O2—S1—O1	119.91 (10)	C1—C6—H6A	119.7
O2—S1—N1	108.45 (10)	O3—C7—O4	124.6 (2)
O1—S1—N1	104.97 (10)	O3—C7—C3	125.4 (2)
O2—S1—C9	108.08 (10)	O4—C7—C3	109.98 (19)
O1—S1—C9	108.68 (10)	O4—C8—H8A	109.5
N1—S1—C9	105.91 (10)	O4—C8—H8B	109.5
C7—O3—Br1^i^	134.06 (17)	H8A—C8—H8B	109.5
C7—O4—C8	117.9 (2)	O4—C8—H8C	109.5
C1—N1—S1	121.14 (16)	H8A—C8—H8C	109.5
C1—N1—H1A	118 (2)	H8B—C8—H8C	109.5
S1—N1—H1A	110 (2)	C14—C9—C10	121.5 (2)
C6—C1—C2	118.7 (2)	C14—C9—S1	119.90 (18)
C6—C1—N1	119.76 (19)	C10—C9—S1	118.60 (17)
C2—C1—N1	121.51 (19)	C11—C10—C9	119.1 (2)
C3—C2—C1	120.81 (19)	C11—C10—H10A	120.4
C3—C2—Br1	119.86 (16)	C9—C10—H10A	120.4
C1—C2—Br1	119.31 (16)	C10—C11—C12	119.1 (2)
C2—C3—C4	119.4 (2)	C10—C11—H11A	120.5
C2—C3—C7	121.8 (2)	C12—C11—H11A	120.5
C4—C3—C7	118.8 (2)	C13—C12—C11	121.9 (2)
C5—C4—C3	120.3 (2)	C13—C12—Cl1	119.4 (2)
C5—C4—H4A	119.8	C11—C12—Cl1	118.8 (2)
C3—C4—H4A	119.8	C12—C13—C14	119.0 (2)
C4—C5—C6	120.0 (2)	C12—C13—H13A	120.5
C4—C5—H5A	120.0	C14—C13—H13A	120.5
C6—C5—H5A	120.0	C9—C14—C13	119.4 (2)
C5—C6—C1	120.7 (2)	C9—C14—H14A	120.3
C5—C6—H6A	119.7	C13—C14—H14A	120.3
			
O2—S1—N1—C1	−47.0 (2)	C8—O4—C7—C3	−179.6 (2)
O1—S1—N1—C1	−176.30 (17)	C2—C3—C7—O3	75.4 (3)
C9—S1—N1—C1	68.80 (19)	C4—C3—C7—O3	−104.3 (3)
S1—N1—C1—C6	64.4 (3)	C2—C3—C7—O4	−105.9 (3)
S1—N1—C1—C2	−118.6 (2)	C4—C3—C7—O4	74.3 (3)
C6—C1—C2—C3	1.4 (3)	O2—S1—C9—C14	5.6 (2)
N1—C1—C2—C3	−175.7 (2)	O1—S1—C9—C14	137.26 (19)
C6—C1—C2—Br1	179.72 (16)	N1—S1—C9—C14	−110.4 (2)
N1—C1—C2—Br1	2.6 (3)	O2—S1—C9—C10	−175.16 (17)
C1—C2—C3—C4	−2.6 (3)	O1—S1—C9—C10	−43.5 (2)
Br1—C2—C3—C4	179.04 (17)	N1—S1—C9—C10	68.8 (2)
C1—C2—C3—C7	177.6 (2)	C14—C9—C10—C11	−1.2 (4)
Br1—C2—C3—C7	−0.7 (3)	S1—C9—C10—C11	179.62 (18)
C2—C3—C4—C5	1.3 (3)	C9—C10—C11—C12	1.2 (4)
C7—C3—C4—C5	−178.9 (2)	C10—C11—C12—C13	−0.3 (4)
C3—C4—C5—C6	1.2 (3)	C10—C11—C12—Cl1	179.35 (19)
C4—C5—C6—C1	−2.5 (3)	C11—C12—C13—C14	−0.6 (4)
C2—C1—C6—C5	1.2 (3)	Cl1—C12—C13—C14	179.75 (19)
N1—C1—C6—C5	178.3 (2)	C10—C9—C14—C13	0.3 (4)
Br1^i^—O3—C7—O4	−27.5 (4)	S1—C9—C14—C13	179.46 (18)
Br1^i^—O3—C7—C3	150.97 (18)	C12—C13—C14—C9	0.6 (4)
C8—O4—C7—O3	−1.0 (4)		

Symmetry code: (i) −*x*+1, *y*+1/2, −*z*+1/2.

### Hydrogen-bond geometry (Å, º)

**Table:**

*D*—H···*A*	*D*—H	H···*A*	*D*···*A*	*D*—H···*A*
N1—H1*A*···O1^ii^	0.80 (4)	2.22 (4)	2.978 (3)	158 (3)

Symmetry code: (ii) −*x*+1, −*y*, −*z*+1.
